# Adaptation to SARS‐CoV‐2 under stress: Role of distorted information

**DOI:** 10.1111/eci.13294

**Published:** 2020-06-13

**Authors:** Konstantin S. Sharov

**Affiliations:** ^1^ Koltzov Institute of Developmental Biology Russian Academy of Sciences Moscow Russia

**Keywords:** COVID‐19, exaggerated information, healthcare system stress, misallocation of resources, population adaptation, social panic

## Abstract

**Background:**

Since the time of global SARS‐CoV‐2 spread across the earth in February 2020, most of countries faced the problem of massive stress of their healthcare systems. In many cases, the structural stress was a result of incorrect allocation of medical care resources. In turn, this misallocation resulted from fear and apprehensions that superseded thorough calculations. A key role in exacerbating the healthcare sector overburdening was played by misleading information on the virus and disease caused by it. In the current paper, we study the situation in Russian healthcare system and advance recommendations how to avoid further crises.

**Materials and methods:**

(a) Surveying the medical personnel (231 doctors, 317 nurses and 355 ambulance medical workers of lower levels) in five hospitals and six ambulance centres in Moscow. (b) Content analysis of 3164 accounts in Russian segment of social networks (VKontakte, Facebook, Instagram, Twitter, Odnoklassniki); official and unofficial media (TV, informational webpages).

**Results:**

We revealed positive‐feedback loop that threatened the sustainability of Russian care sector. The main knot was occupied by incorrect/exaggerated media coverage of COVID‐19. General public scared by misinformation in media and social networks, started to panic. This negative social background undermined the productivity of a significant part of medical workers who were afraid of COVID‐19 patients.

**Conclusions:**

The most serious problems of Russian healthcare sector related to COVID‐19 pandemic, were informational problems. The exaggerated information on COVID‐19 had big negative influence upon Russian society and healthcare system, despite SARS‐CoV‐2 relatively low epidemiological hazard.

## 1 INTRODUCTION

### 1.1 Focus

Professor John Ioannidis clearly demonstrated that a threat of false or unconfirmed information on COVID‐19 might endanger almost all scopes of life.^1^ In chapters 6 and 7 of his communication, he drew our attention to a hazard of wrong, highly biased allocation of resources. In Russia, with already 232 243 persons tested positive for SARS‐CoV‐2 (official Russian governmental data as of 12 May 2020),^2^ we currently face such a situation in healthcare sector. In our article, we study negative social impact of exaggerated information about COVID‐19 on Russian society and healthcare system.

### 1.2 Problematic overview

Since the beginning of SARS‐CoV‐2 outbreak, many states, including European countries, faced massive stress for their healthcare systems. Such situation has been recently reported for Italy,^3^ Austria, Germany, Switzerland,^4^ the Netherlands,^5^ Spain,^6^ France,^7^ USA,^8^, ^9^ India,^10^ China,^11^ and Turkey.^12^ A thorough analysis of informational coverage of the virus spread shows that distorted information was one of the major causes of excessive, inappropriate and often inconsistent actions in public and healthcare policy.

A few recent examples are instructive. In UK, the population stress related to exaggerated news on COVID‐19, resulted in the increased number of heart attacks through elderly people.^13^ In Germany, Robert Koch Institute continues to issue its daily reports with a warning assessment message “The RKI currently assesses the risk to the health of the German population overall as **high** and as **very high** for risk groups”.^14^ Such evaluations were revealed to cause large anxiety and mental stress among nursing staff in Germany, particularly in Berlin.^15^ In Italy, especially in Lombardy, many people got scared to call the emergency and potentially be hospitalized due to the risk of COVID‐19 contraction in a hospital.^16^ Consequently, many COVID‐unrelated cases that had required immediate interference, remained unobserved by medical personnel.

Sucharit Bhakdi^17^ and Daniel von Wachter^18^ drew our attention to obvious harms of uncritical treating the information about COVID‐19 by politicians, administration and society in Germany as well as disproportional public and healthcare policy measures. In January 2020, Wolfgang Sassin emphasised the inappropriateness of German administrative steps aimed at mitigating epidemiological and demographical hazards.^19^, ^20^

Therefore, the lessons learned from the Russian negative and positive experience with COVID‐19 containment may have heuristic value for healthcare sectors of other European countries, since similar challenges are now faced in almost every country.

## 2 MATERIALS AND METHODS

### 2.1 Survey

We surveyed medical care personnel of different levels by obtaining written responses to our questionnaire. The medical staff in question works in five Moscow hospitals transformed to infectious disease infirmaries for treating only COVID‐19 patients, and six ambulance centres. The data obtained were treated semantically and afterwards statistically. 231 doctors, 317 nurses and 355 ambulance medical workers of lower levels (medical assistants) have been surveyed in total.

### 2.2 Content analysis

Content analysis was used as a technique of studying social reaction to COVID‐19 exaggerated information in Russian segment of social networks VKontakte, Facebook, Instagram, Odnoklassniki, Twitter. 3164 accounts have been analysed. Content analysis of Russian media (seven TV news broadcasts and eight talk shows on primary and secondary TV channels, official governmental websites on counteracting COVID‐19) was also performed.

### 2.3 Time range

Collecting the primary data was carried out since 2 March to 10 May 2020.

### 2.4 Software

**Figure 1:**
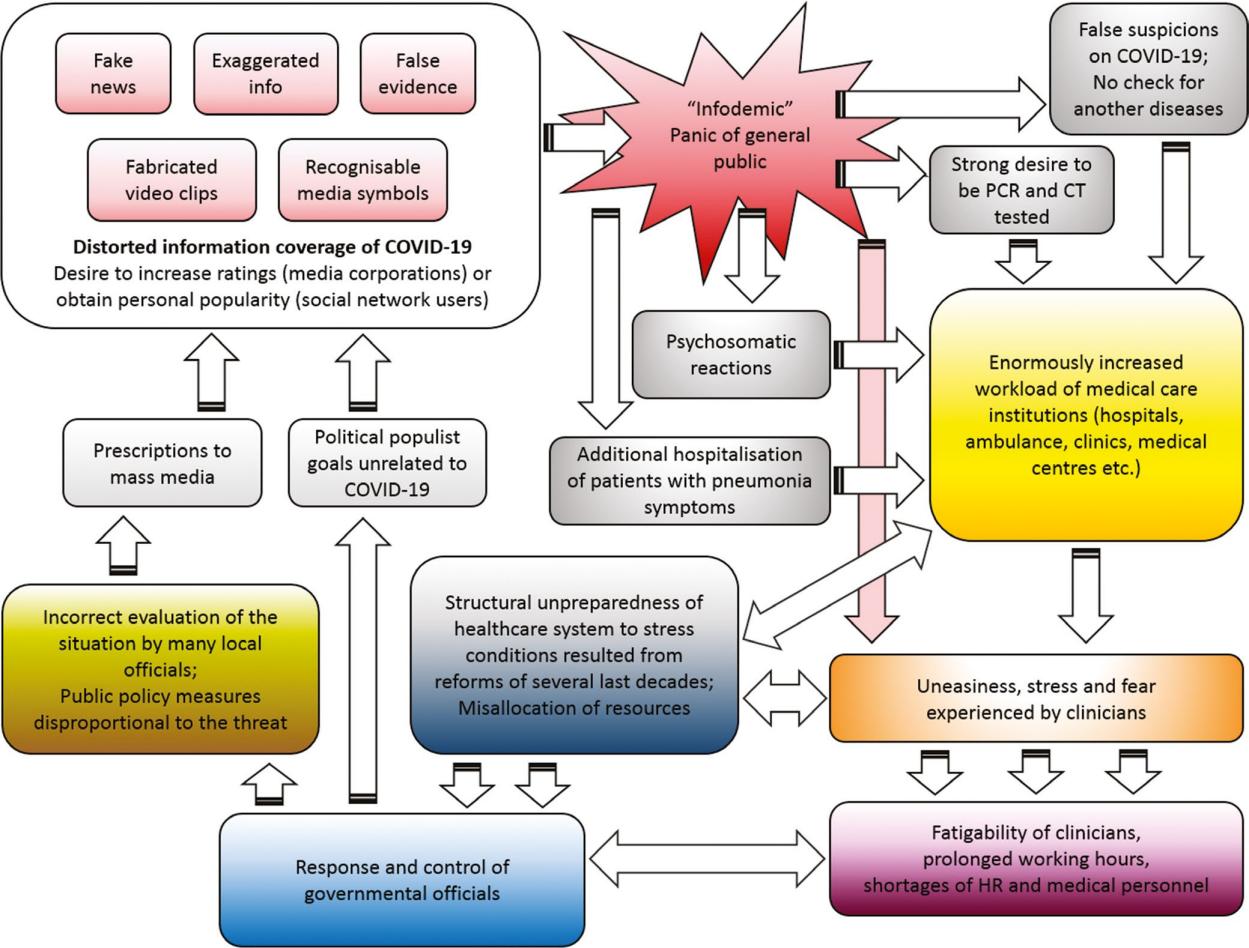
Positive‐feedback loop that causes current overheating of Russian healthcare system. Acronyms used: PCR—Polymerase chain reaction (a most common technique for the virus detection); CT—Computer tomography (the lungs state analysis); HR—Human resources

MS Visual Studio 2010 (C#) was used for analysing the data obtained in the survey..NET Framework 4.5 tools were used to write a proprietary C#‐coded module for social networks content analysis. MS PowerPoint 2013 was used for creating the Figure 1.

### 2.5 Ethical guidelines

Reporting of the study conforms to broad EQUATOR guidelines.^21^

## 3 RESULTS AND DISCUSSION

### 3.1 Results of surveying medical workers

In the survey, it became clear that 64.2% of polled doctors and 82.2% of polled medical personnel of lower levels (medical assistants and nurses) (hereinafter the percentage values are produced from the total number of the surveyed, and confidence interval is 95%) were experiencing stress and constant fear to become infected. 47.1% of clinicians with at least MD degree and 70.6% of medical personnel of lower educational levels responded in the poll questionnaire that this fear mainly proceeded from media sources, including TV news and social networks, not from their daily hospital experience. Many of respondents (36.5 and 30.1%) confessed that they probably would have performed their professional duties better, had it not been for their stress and fatigue.

### 3.2 Results of content analysis of personal posts on COVID‐19 in social networks

Our research demonstrated that the role of using social networks and electronic gadgets in spreading panic throughout Russian hospital clinicians was enormous. Moreover, we concluded that stress and anxiety within medical care were mainly caused by the overall highly nervous social background. This background, in turn, was instigated by false or unproven information on the epidemic.

The content analysis of social network personal accounts of Russian‐speaking audience with at least one network post concerned with the current epidemic or related matters and written in time interval 25 February—10 May 2020, showed that 81.4% of the authors or disseminators of the posts in question exhibited high degrees of anxiety and even panic brought about by distorted, exaggerated or false information on COVID‐19 disease and its causative agent.

### 3.3 Major venues of producing misinformation or distorted information about COVID‐19 in Russia

In our analysis, we isolated seven major venues of producing such information.

1. Social networks (Russian sectors of Facebook, Instagram, Twitter, as well as Russian networks VKontakte and Odnoklassniki) are the primary places of creating, replicating and transmitting dubious, false and unconfirmed information on the epidemic. Shocking “news” on COVID‐19 are appearing and being forgotten almost every day, but sometimes they cause dramatic effects on general public. We observed that in the atmosphere of permanent stress and fear, an essential part of Russian social network users (41.9%) tended to believe uncritically in any unconfirmed “facts” about the disease and SARS‐CoV‐2, whichever fantastic they might seem.
2. World Health Organisation (WHO) official websites with their real‐time statistics,^22^ cause considerable negative impact on Russian social network users. The current epidemic is regarded by many as an online real‐time show anyone can observe in a 24 × 7 mode. SARS 2002‐2004 and H5N1 avian flu 2004‐2008 outbreaks passed almost unnoticed by the Russian society, whereas SARS‐CoV had at least twofold fatality rate in comparison with SARS‐CoV‐2^23^ and H5N1 flu at least 10‐fold.^24^ We see the main reason of it in a much lesser presence of Internet and electronic gadgets in Russia in 2003‐2004 in comparison with 2020 (for general public as well as medical workers). WHO’s “Confirmed deaths” counter continuous ticking negatively influences audience. Multiple comments in Russian social networks show that almost nobody pays heed to the fact that, on average, a seasonal flu causes greater net fatality every year than COVID‐19 caused thus far. We revealed that WHO’s “Confirmed deaths” counter was interpreted by Russian public unambiguously as an evidence of the virus utmost danger to the population.
3. Daily news on major Russian TV channels are constructed in such a way that they demonstrate the rate of “those who got ill (infected, contaminated and stricken) today from COVID‐19” and “perished today form COVID‐19.” Even disregarding the dubious nature of the numbers “Confirmed cases” and “Confirmed deaths” introduced by WHO,^18^ we observed that using such phraseology created an alarming feeling of war communiqués. To perish is to be killed in action. Therefore, the audience watching TV news, starts to panic as it were a wartime (57.3% of analysed social network accounts contained various ideas about parallels between COVID‐19 epidemic and alleged World War III). Additionally, the collocation “stricken with COVID” was identified by 62.3% of social network users as an indicator of very high SARS‐CoV‐2 contagiousness.
4. Most of Russian TV talk shows are staged in a similar way. A persistent narrative about “how many people were killed by the virus heretofore” remains their main topic. The only commendable example of a contrary approach is Alexander Gordon's TV show “Doc Talk” where the host and his guests have been trying to find real answers and soothe media psychosis common for Russian TV.
5. The official website of the Russian Official Headquarters on Control and Monitoring of Coronavirus Situation^2^ is little informative. A novel and uncommon word “coronavirus” whose meaning was not duly explained by officials of Ministry of Health of Russian Federation, impacted on both Russian society and medical workers negatively. If there had been a clear and timely explanation of the fact that there are four coronaviruses that caused dramatic outbreaks in the very recent past but now transformed to aetiological agents of seasonal acute respiratory infections (human coronaviruses HKU1, OC43, 229E and NL63),^25^, ^26^, ^27^ a large part of anxiety, stress and fear would have been probably avoided. In addition, common naming SARS‐CoV‐2 “an unprecedented survival challenge” by Russian media did not mollify the general fears. No mass media notified broad society and medical care workers that the “once‐in‐a‐century virus,” the “virus not encountered thus far” in reality shares around 80% of genetic sequence with SARS‐CoV that caused the epidemic of 2002‐2004.^28^, ^29^, ^30^, ^31^ The results of our survey showed that 48.2% of doctors and 93.3% of medical workers of lower levels (not virologists) had no idea about SARS‐CoV‐2 being just another representative of human coronaviruses subfamily till the end of the main survey, 26 April 2020. Of 3164 inspected social network accounts, no user exhibited signs of such knowledge. The majority of people use mass media rather than scientific papers, as informational resources. And mass media provide mainly distorted and biased coverage, pouring additional oil on the flames.
6. Social advertisement on TV channels exploiting the children's services was insistent on the major Russian TV channels (1TV, Russia TV, TVC and NTV) in time range 22 March—20 April 2020. Clips with children of 4‐8 years appeared constantly with messages to imagined grandmothers and grandfathers. This caused anxiety in 17.4% of doctors and 24.2% of medical personnel of lower levels. 18.2% of social network users also demonstrated signs of nervousness related to these clips.
7. Internet search engines most commonly used in Russia, Yandex and Google, paint a web browser page in alarming reddish colours, provide unnecessary disquieting recommendations to a person what he/she should do, and show WHO misleading indicators, on every mentioning “NCoV,” its synonyms or any related matters in a search line. According to our estimations, this is a cause of raising discomposure of 12.8% of social account users.

### 3.4 Persistent media symbols related to COVID‐19 with misinformation messages

In our research, a few persistent media myths created with the help of well‐recognisable symbols (several of them were obviously staged‐produced) have been demonstrated to lead to the highest level of alarming the Russian network society, including medical workers. These symbols are the following:

- heaps of coffins;
- mass burials and overloaded crematoriums;
- medical personnel in Kommunarka hospital (the first hospital that was lately built near Moscow exclusively for treating COVID‐19 patients) wearing spacemen‐like anti‐plague medical protective costumes with gas masks;
- tears of the infected, deemed to reflect severe physical sufferings;
- empty streets demonstrated from distant perspectives;
- closed or empty Christian temples.

These symbols have little to do with the containment of the disease, but, as we found, they cause large negative impact on social network users in Russia.

### 3.5 Positive‐feedback loop of Russian healthcare sector overheating

We revealed the following positive‐feedback loop (Figure 1). This loop may be regarded as the main reason of Russian healthcare system overheating.

In Russia, we currently observe two very dangerous places in this loop. First, there was no initial calculation of need in ventilator intensive care units (ICUs) and general wards for COVID‐19 patients by authorities. Fear and distorted information influenced Russian healthcare bureaucrats and a number of other governmental officials who issued the directives to transform excessive amount of hospitals to COVID‐19 infirmaries. Only in Moscow, more than twenty hospitals, including highly specialized ones such as cardiovascular, oncological and cerebrovascular, stopped to admit non‐COVID patients in March 2020—early April 2020. Additional twenty‐four hospitals were transformed later.^32^ This amount may be excessive. As a result, many patients were not treated properly by required specialists, who were ordered to focus on COVID‐19 patients instead. Many serious non‐COVID patients were entrusted to general medical workers with specialisation in different areas of medicine. In addition, too much reliance has been initially put upon mechanical ventilator treatment, whereas only critically ill persons with acute respiratory distress syndrome and respiratory failure must be treated by mechanical ventilators lest a damage to a patient should exceed a potential good.^9^ Second, constant media panic brought about an enormous level of calls of general public to ambulance and hospitals. Many people in Russia continue to require to be hospitalized with mildest symptoms of acute respiratory infection even thus far (as of 12 May 2020). Healthcare system usually satisfies such applications, while non‐COVID persons who are critically ill may remain without an appropriate and timely medical help at all due to overburdening the care sector by large amount of people in anxiety.

We suggest the following measures to reduce the current stress of medical workers and overheating of healthcare sector:

- Regular demonstrating full mass‐testing statistics in hospital bulletins/ communiqués, media and social networks instead of simple replicating WHO metrics. It will clearly show low contagiousness and fatality rate of SARS‐CoV‐2 (currently in Russian population, there are 3.94% infected persons, and within this cohort, there are 0.91% deceased persons: 2116 of 232 243 people, as of 12 May 2020^2^ — this is even ascribing every deceased person with SARS‐CoV‐2 tested to COVID‐19, without any further investigation what reason(s) might really cause death);
- Revealing deceptive nature of well‐recognisable media symbols created specially to cause shock and stress (coffins, anti‐plague medical costumes, mass burials, tears, etc);
- Political initiatives aimed at curbing media psychosis.

## 4 CONCLUSIONS

The most serious and urgent problems of Russian healthcare sector related to SARS‐CoV‐2 are structural and informational problems. The exaggerated and distorted information on COVID‐19 has big negative influence upon Russian society and healthcare system, despite SARS‐CoV‐2 relatively low epidemiological hazard.^1^, ^33^, ^34^, ^35^

Theoretically, in the future we may face a much more dangerous virus that will present a real threat (e.g. a virus with fatality of Ebola and contagiousness of chickenpox). What steps should be taken to avoid similar unpreparedness of the healthcare sector in such a case? We may propose the following measures relevant for both Russia and other countries:

1. Implementing effective ad hoc search‐and‐isolate techniques of virus containment aimed at immediate revealing virus carriers instead of imposing lockdown measures on the entire population that violates constitutional and democratic rights of citizens and results in economies disruption, “empty” money printing and budget overloads.
2. Stricter and more effective epidemiological surveillance in airports, international railway stations and border control premises.
3. Technical preparedness of infectious disease branch of medical care system.
4. Thorough distinguishing between those patients who really need hospitalisation (seriously ill) and those who may be treated at home (asymptomatic or mild symptomatic patients).
5. Allowance of capacities of individual protective units (IPU) for medical personnel and general public. As of late April 2020, in some Russian hospitals transformed to COVID‐19 infirmaries, medical workers had to buy third parties’ IPUs at their own expense (a similar situation of shortages of IPUs was reported for Italy by Pecchia et al^36^).
6. Control of informational coverage of an epidemic on TV media sources. Providing more comprehensive governmental reports on epidemiological situation. These reports shall be made freely available for general public and medical workers. Avoidance of making an online real‐time show of an epidemic. Presenting materials on an epidemic in such a way that would not boost panic in society and among clinicians.
7. Preparing up‐to‐date informational press releases in hospitals, ambulance and medical testing centres with relevant information about a pathogen.
8. Fixing persistent myths, misleading media images and video clips that are going viral in the web, with further professional analysing their mythical character by virologists and other expert researchers.

Fear, panic, disproportional public policy measures and consequent misallocation of resources in medical care system, resulting from the uncritical acceptance of exaggerated and unconfirmed information on SARS‐CoV‐2, should be contained even faster than the virus itself, if we would like to succeed in the struggle against the disease and continue to be called free and democratic society.

## CONFLICTS OF INTEREST

The author declares no conflicts of interest.
